# Social inattentional blindness to idea stealing in meetings

**DOI:** 10.1038/s41598-024-56905-6

**Published:** 2024-04-05

**Authors:** Theodore C. Masters-Waage, Zoe Kinias, Jazmin Argueta-Rivera, Dillon Stewart, Rachel Ivany, Eden King, Mikki Hebl

**Affiliations:** 1 INSEAD, Paris, France; 2 University of Houston, Houston, USA; 3 Western University, London, Canada; 4 Rice University, Houston, USA

**Keywords:** Human behaviour, Psychology

## Abstract

Using a virtual reality social experiment, participants (N = 154) experienced being at the table during a decision-making meeting and identified the best solutions generated. During the meeting, one meeting participant repeated another participant’s idea, presenting it as his own. Although this idea stealing was clearly visible and audible, only 30% of participants correctly identified who shared the idea first. Subsequent analyses suggest that the social environment affected this novel form of inattentional blindness. Although there was no experimental effect of team diversity on noticing, there was correlational evidence of an indirect effect of perceived team status on noticing via attentional engagement. In sum, this paper extends the inattentional blindness phenomenon to a realistic professional interaction and demonstrates how features of the social environment can reduce social inattention.

## Introduction

A perplexing feature of human experience is our inability to notice salient objects or events while engaged in another task. In a series of striking experiments, researchers showed that while engaged in a simple counting task individuals failed to notice a person in a gorilla suit beating their chest, a woman holding an umbrella, or a loud electronic sound^1,2^. This phenomenon is termed inattentional blindness, and it demonstrates that although our experience of the world may feel rich and detailed—hearing, tasting, smelling, and feeling the world simultaneously^3,4^—in reality, the human brain is only processing a minutiae of the sensory information from the environment at any one time^5,6^. These findings have had a profound effect on our understanding of the human brain^5^. However, if inattentional blindness is as dramatic as these laboratory experiments suggest, it must also have profound implications for the social dynamics that play out in everyday work interactions.

The workplace can be a hectic environment in which individual employees are required to juggle numerous work tasks simultaneously^7^. Based on research on inattentional blindness, this combination of task-focused work and an information-rich environment, creates a high likelihood that when employees’ attention is engaged in one task, they are unlikely to notice task-unrelated (but important) stimuli in their environment^1^. In the real world, this task-unrelated information is not as peculiar as a man in a gorilla suit walking across one’s field of vision but the consequences can be more impactful. For example, notable organizational failures have resulted from individuals failing to notice readily available and seemingly obvious information, including NASA engineers not noticing the evidence that the O-Rings would fail on the Space Shuttle Challenger, financers not noticing Bernie Madoff’s Ponzi scheme, and regulators not noticing Enron’s impossible account records^8^. These failures highlight the critical role that noticing plays in organizations and begs the question of what information we are inattentive to in our social environments at work. This paper examines this question at a foundational level by exploring whether people notice an important social event occurring within an organization or if they exhibit *social inattentional blindness*.

The social event this paper focuses on is idea stealing, which is defined as someone pursuing, or taking credit for, an idea that is perceived to be owned by another^9^. Specifically, we examine whether team members in an idea generation meeting notice when one team member steals another’s idea. Idea generation is a critical function of organizational groups, allowing them to solve existing problems and create future innovations^10–12^. Therefore, individuals receiving credit for generating a good idea can yield reputational benefits, potentially leading to career advancement and financial rewards. Therefore, there can be a personal motivation for individuals to steal others’ ideas. However, the reputational cost of being noticed to steal an idea is high, with one study finding that people viewed idea stealers as being more unethical than people who stole money^9^. Nevertheless, idea stealing is prevalent within organizations, with a 2015 poll of 1000 British workers finding that 46% of workers said that colleagues had stolen their ideas to make themselves look better, and 20% admitted to stealing ideas themselves^13^. This begs the question, if idea stealers are so severely punished, then why are so many employees engaging in this behavior? We theorize that it is due to other employees' social inattentional blindness to idea stealing in meetings.

Research using the “Who Said What?” paradigm has demonstrated source-tag confusions in memory, in other words, meaningful errors in linking the sentiment shared in a meeting with the person who said it^14^. A conclusion from this research is that it requires additional effort and mental processes to attend to the information being shared *and* the person who is sharing that information^15,16^. This is critical because in an idea generation meeting the primary goal of team members is to generate ideas, as well as to listen to and evaluate the ideas/information shared^4^, not necessarily to keep account of who is sharing that information. Further, on top of attending to what is shared, individuals must also consider the “pros and cons” of multiple ideas simultaneously making this a demanding task^17^. Given the inherent limits of human cognition, we argue that by focusing on the task of information processing observers devote fewer mental resources to noticing who said what idea, creating oversights in assigning credit. This means that while in most cases the people who present themselves as generating ideas are the owners, when this assumption is violated because someone steals another person’s idea, we hypothesize that a significant portion of individuals will not notice that the idea was stolen. We term this phenomenon social inattentional blindness.

Expanding the inattentional blindness paradigm to the social/organizational domain allows us to examine how aspects of the social environment affect noticing. Past research has found individuals with a higher working memory capacity, are less susceptible to inattentional blindness^18^. Further, mindfulness interventions that increase present moment attentiveness have been shown to reduce susceptibility^19^. Therefore, we hypothesize that features of the social environment that cue individuals to be more attentive might in turn reduce social inattentional blindness. Specifically, following from theory and evidence on diversity improving group decision-making and reducing groupthink^20^ we focus on how increasing the demographic diversity of the group (while keeping the demographics of the idea sharer and stealer constant) reduces inattentional blindness.

**Figure 1:**
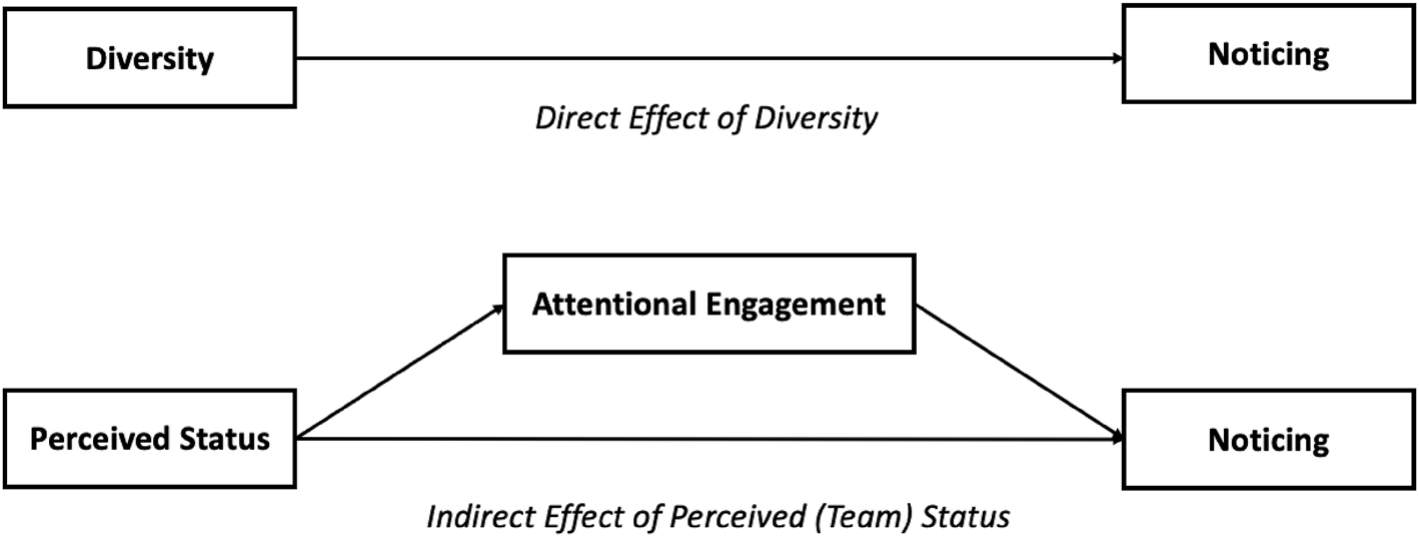
Theoretical models tested in this paper, first the “direct effect of diversity” on noticing and second the “indirect effect of perceived (team) status” on noticing via attentional engagement.

Organizational teams research has found evidence for performance benefits of diversity^20,21^. This is attributed to a variety of mechanisms, including that team members prepare more thoroughly and are more engaged when in diverse teams^22^. By being more engaged, we argue that individuals in diverse teams are also more likely to be attentive to their environment and thus be less susceptible to social inattentional blindness (see Fig. 1 for a pictorial representation of this hypothesis).

More broadly, we also examine how individuals' perceptions of team status might affect attentiveness in the meeting. Past research has linked status and attention within meetings, with group members who have more status within groups capturing more attention^23,24^. For example, participants observing a video recording of a group meeting pay more attention to the faces of the group members who were rated within the group as having higher status, as measured through eye tracking^24^. We propose that status increasing attention to specific members of a group can translate from the individual to the group level, such that groups perceived as having higher status also receive more attention than groups perceived as having lower status. Thus, we hypothesize that individuals are more attentive in teams they perceive to be higher status, which in turn will reduce social inattentional blindness (see Fig. 1).

In sum, by extending the inattentional blindness phenomenon to the social domain using VR, we develop this body of knowledge in several ways. First, we investigate the role of inattentional blindness in a real-world environment (idea generation meeting) and focus on a common and meaningful phenomenon (idea stealing). This contrasts past work which has focused on inattention to unusual events, such as a person in a gorilla costume beating his chest^25,26^, that are unlikely to occur in real-world settings. Second, we explore whether inattentional blindness occurs in multisensory environments (visual and auditory), not just purely visual^25^ or auditory^27^. Third, we examine a new research question, which is how the social environment individuals are in (i.e., the diversity of their team and their perceptions of the team’s status) affects susceptibility to inattentional blindness. This goes beyond past work focusing on how individual-level factors affect susceptibility^18,19^.

## Virtual reality social experiments

This study employs a virtual reality based social experiment to examine how individuals pay attention to a social event. Classically, social experiments have been conducted using confederates (trained actors) to artificially create a social situation^28^. Many iconic psychological experiments have used this approach—e.g., Milgram’s obedience study^29^ and Asch’s conformity study^30^—but they present numerous challenges to researchers. Foremost, even if well trained, experimenters cannot perfectly control variability in confederates’ behaviors across participants. Further, from a practical perspective, social experiments place a high demand on resources which makes these experiments costly in terms of time, space, personnel, and money, thus limiting the feasible sample size that can be collected^28^. Virtual reality provides an innovative solution to this challenge^31^.

Virtual reality (VR) is an emerging experimental research methodology in psychological science^31^. The goal of the method used in this study is to generate a simulated environment with the benefits of being realistic and controlled. By immersing participants in a virtual environment, VR experiments are able to increase the level of presence participants experience, relative to paper-based or video-based stimuli, while preserving full experimental control^32^. This maximization of the control-realism tradeoff in VR is particularly advantageous for attention-based research, which requires a high degree of control, as even a minor change to the environment (e.g., room brightness, a person sneezing, a sideways glance) can significantly affect where participants allocate their attention^33,34^, and a high degree of realism, as feeling physically present in an environment significantly affects how people attend to social information^35^. Further, VR experiments also provide participants with the agency over where to focus their attention in an environment, creating a more realistic representation of the competing demands on attention that individuals experience in the real world, relative to videos that offer only the “director’s lens” on where to focus attention.

Within VR research there are different approaches. An important distinction to be made is between VR using a computer-generated environment in which individuals can move around and interact with their environment (i.e. six-degrees of freedom^36^) vs. VR using a video-generated environment displaying 360-degree video in which individuals can observe a live-action environment from a stationary point (i.e. three-degrees of freedom^36^). The choice between these two approaches balances trade-offs, e.g., computer-generated environments provide the participant with more freedom, but 360-videos are more realistic. In this paper we use the latter option, due to the increased realism and given that the research question does not require participants to interact with their environment.

Participants experienced the VR social experiment in which four actors played the parts of employees in a decision-making meeting through a head-mounted display (HMD), specifically a Pico device. This device played the 360-video which was separated into different scenes and multiple-choice questions were spliced in between them, which participants responded to using a “select” button on the side of the headset (for more details on the VR design see the procedure section of the methods). One of the scenes includes a case of idea stealing, which the participants witness as an observer. We purposefully left the intention of the idea stealer ambiguous, i.e., whether the idea stealer intentionally or unintentionally stole the idea. This is because the phenomenon of idea stealing often occurs with ambiguous intentions and where the observers (i.e., participants) do not know the idea stealer’s intentions. Participants were assigned to one of two groups. In both groups the content of the meeting (i.e., the script the actors were following) was identical, however the gender and race of the actors changed. In the homogenous group (i.e. low diversity) all actors were white men. In the diverse group there were two white men, one white woman, and one black man: note, the two white men were the original idea sharer and idea stealer to keep the identity of those actors the same across conditions. These two conditions simultaneously manipulate group diversity in terms of gender *and* race. We also acknowledge that separate conditions examining the effects of gender *or* racial diversity would also be interesting and highlight this in the discussion section, however, such a research question would go beyond the intention of this study which was to examine the effect of diversity (in general) on social inattentional blindness.

## Results

Descriptive statistics and correlations for all study variables are provided in Table 1. The script for the location decision, in which the idea stealing occurs, is presented in Supplementary Appendix 1 with the key sentiments highlighted. In real-time, the idea stealing occurred roughly 90 seconds after the original idea was shared. Additional analyses of the effects of participant demographics on noticing and a set of exploratory analyses on the indirect effect of team diversity on noticing via a serial mediation through perceived team diversity, perceived team status, and attentional engagement, are included in the supplementary materials.

**Table 1:** Descriptive statistics and correlations between study variables.

	Mean	SD	1	2	3	4	5	6
1. Condition	0.50	0.50	–					
2. Diversity	2.24	1.32	0.64***	–				
3. General attentiveness	5.35	1.34	− 0.09	0.02	(0.94)			
4. General inattentiveness	4.04	1.31	0.03	0.00	− 0.67***	(0.74)		
5. Perceived team status	11.11	0.99	0.08	0.21**	0.27***	− 0.17*	(0.85)	
6. Noting (idea sharer)	0.30	0.46	0.07	0.00	0.17*	− 0.18*	− 0.09	–
7. Credit allocation (idea sharer)	0.30	0.46	0.15^+^	0.13	0.12	− 0.14^+^	− 0.02	0.79***

Condition was coded as 1 “Diverse” and 0 “Homogenous”, Diversity measure, general attentiveness, general inattentiveness, and perceived team status responses were coded as 1 “Strongly Disagree” to 7 “Strongly Agree”, noticing (idea sharer) was coded as 1 “Participants correctly noticed the original idea sharer” or 0 “they did not”, credit allocation (idea sharer) was coded as 1 “Participants correctly credited the original idea sharer” or 0 “they did not”. Alpha coefficients are in parentheses. ***p < 0.001, **p < 0.01, *p < 0.05, ^+^p < 0.10.

### Social inattentional blindness

The first set of analyses examined the extent to which participants noticed the idea stealing occurring in the meeting. The “riverside idea” that was stolen, was chosen by 99.33% of participants as the best idea. However, when asked who shared the “riverside idea” first, only 30.20% of participants correctly identified the original idea generator. Consistent with the idea that noticing who shared the idea first also determined who got credit, 29.53% of participants gave the original idea generator credit (note, one participant noticed he shared the idea first but did not give him credit). Using a binomial test, we found that participants did not perform better than chance (25%) on correctly noticing who shared the idea first (p = 0.155).

Building on this, there was evidence that the team member who stole the idea benefited from doing so. A total of 38.93% of participants believe that the idea stealer was the person who said the idea first and 42.28% of participants gave the idea stealer credit. The remaining participants (29.53%) believed that one of the other two team members (who neither originally shared the idea nor stole it) had said it first, showing the high level of inattention to noticing idea stealing.

Next, based on previous work suggesting that individuals with increased attentional awareness were less susceptible to inattentional blindness^18^, we examined whether participants who reported higher levels of general attentiveness after the meeting were more likely to have noticed the idea stealing. To account for a possible demand effect, attentiveness was measured both in the positive sense (how attentive were you?), and the negative sense (how inattentive were you?). These measures were strongly correlated (r = − 0.67, p < 0.001), and results were the same using both measures. Using logistic regression, we regressed whether participants correctly noticed who shared the idea first on self-reported attentiveness. Participants who reported higher levels of general attentiveness in the meeting noticed idea stealing more frequently (b = 0.31, SE = 0.16, p = 0.048), and those who reported lower levels of general inattentiveness also noticed idea stealing more frequently (b = − 0.30, SE = 0.14, p = 0.033).

This study also sought to measure attentiveness using thought probes, as done previously^37^. These thought probes captured the extent to which individuals were mind wandering (coded as “1”) or not (coded as “0”) in real time throughout the meeting, sampling participants every 22 to 28 seconds. Mind wandering during the idea generation segment of the meeting did not predict noticing idea stealing (b = 0.19, SE = 0.18, p = 0.285), with the trend actually being in the opposite direction. The relationship between mind wandering in the three segments of the meeting before the idea generation segment and noticing who contributed the idea was in the expected direction (i.e., more mind wandering leads to less noticing), however, the result was only approaching significance (b = − 0.12, SE = 0.07, p = 0.058).

### Effect of social environment on social inattentional blindness

First, we examined whether team diversity affected social inattentional blindness. Across the two conditions (diverse vs. homogenous group), 33.33% of participants noticed the idea stealing in the diverse group, whereas 27.03% noticed it in the homogenous group, however, this difference was not statistically significant (t(147) = 0.83, p = 0.405). For credit assignment the difference was larger, with 36% of participants giving the credit for the idea to the original idea generator in the diverse group and only 22.97% in the homogenous group, with this difference approaching significance (t(147) = 1.75, p = 0.082). Further, there was no direct effect of team diversity on general attentiveness (t(146) = − 1.04, p = 0.298), general inattentiveness (t(146) = 0.37, p = 0.707), or mind wandering during the meeting (t(146) = − 0.84, p = 0.400). In sum, there was not a statistically significant main effect of diversity on noticing or credit assignment.

**Figure 2:**
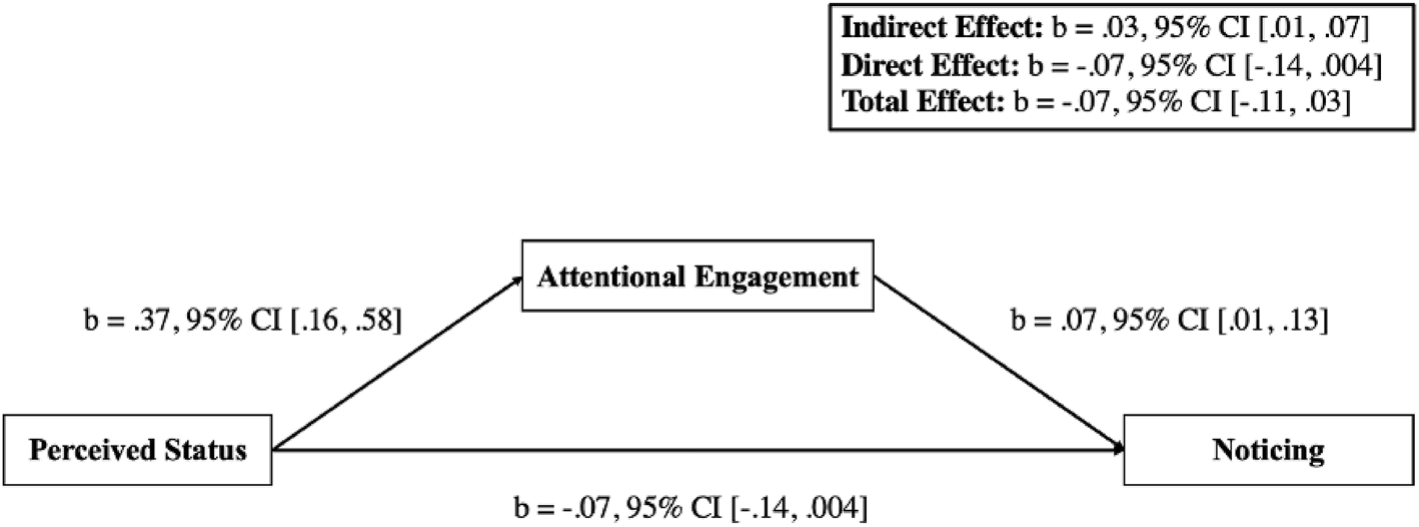
Results from the mediation model tested in this paper, to examine the indirect effect of perceived team status on noticing.

Next, we assessed whether participants’ perceptions of team status affected social inattentional blindness via attentional engagement. Analyses, using 10,000 bootstrapped samples, found no statistically significant direct effect of perceived status on noticing that the original idea generator shared the idea first (b = − 0.07, 95% CI [− 0.14, 0.004]), nor was there a significant total effect (b = − 0.07, 95% CI [− 0.11, 0.03]). However, mediation analyses did find evidence of an indirect effect of status on noticing via increased attentional engagement (indirect effect (10,000 bootstraps): b = 0.03, 95% CI [0.01, 0.07]), such that perceiving a team to be higher status increased noticing (see Fig. 2). Interestingly, while the indirect effect was positive the direct effect of status on noticing was in a negative direction though not significant, which may suggest there are contrasting mechanisms through which status affects noticing. Finally, given there was an effect of status but no direct effect of diversity, on an exploratory basis, we examined whether there was an indirect effect of diversity on noticing idea stealing via perceived (team) status, these analyses are reported in the supplementary materials and find a significant (serially mediated) effect.

## Discussion

This paper provides a compelling answer to why—despite being viewed extremely negatively by others^9^—idea stealing is still common in organizations^13^: people rarely notice it. Results found extreme levels of social inattention to idea stealing in a VR meeting simulation, with only 30% of participants noticing the idea stealing, meaning that people performed no better than chance in identifying who shared the original idea. Further, given that 99% of participants identified the correct solution, this finding shows that participants were paying attention but, consistent with work on the who-said-what paradigm, it appears that individuals process the idea content and who said the idea separately. Moreover, by focusing on the task of idea generation, individuals become inattentive to the source of these ideas.

The extremity of this finding is notable as it demonstrates that even in highly realistic scenarios, individuals still display inattentional blindness to an unexpected event^1^. Further, the failure to notice occurs even though the unexpected event is visually and auditorily observable, and socially meaningful. In doing so, this paper begs the question, if individuals are barely noticing acts as salient as stealing an idea, how many other more subtle unethical acts do employees not notice?

Beyond demonstrating the social inattentional blindness phenomenon, this paper also identified how the social context affects noticing. First, this paper did not support the a priori hypothesis that team diversity would affect noticing, although supplementary analyses did find an indirect effect of the diversity condition (see Supplementary Materials). However, consistent with past work on status capturing attention^23,24^, results from correlational analyses showed that individuals who perceived the team to be of higher status also reported higher levels of attentiveness in the meeting. Given that individuals who were more attentive in general in the meeting were more likely to notice idea stealing, this meant that perceived team status affected inattentional blindness indirectly via increased attentiveness. This finding suggests that perceived status also has an attention capture effect at the team-level. Future research can examine how increased attentiveness in higher status teams has a broader effect on team performance.

Connecting our findings with prior research on attention to high status individuals within groups^23,24^, we see two new insights related to group dynamics, both of which we encourage further research to directly investigate. Although our own data do not allow for such analyses, as we measured status at the group rather than individual level, we encourage future research along these lines. First, it is possible that group members can steal the ideas of lower status group members with lower risk of others observing this. Relatedly, to the extent that original idea generators are attracting attention in meetings due to being high status or otherwise distinctive, this could lead group members and observers to credit them more heavily.

This paper opens intriguing avenues for future research on the social dynamics of idea generation. Idea generation is a key process in any organization and in particular in creative problem solving^38^. As demonstrated in this experiment, individuals were very good at identifying the idea that the team collectively liked, with 99% of participants choosing the riverside location. However, individuals performed at chance in crediting the riverside idea to its original sharer. While this misallocation is likely to negatively affect the original idea sharer, as they will not receive the credit for their idea, the team's collective inattentiveness to who shares what idea may increase the collective ownership of ideas by the team, which has been linked to higher team performance^39^. Therefore, at the team-level, social inattentional blindness may be a beneficial process, even if at the individual-level it means specific people are less likely to receive credit for their ideas. Further, if individuals are focusing attention on who to assign credit to for each idea, they may devote less attention to identifying the best idea.

That said, given the reputational cost of idea stealing, in idea generation meetings a more harmonious way of repeating ideas and generating collective ownership would be amplifying behaviours^40^. It can be difficult to distinguish between idea-stealing behaviours (i.e., taking credit for an idea that is perceived to be another person’s^9^) and attempted amplifying behaviours (i.e., public endorsement of another person’s contribution, with attribution to that person^40^). Regardless of intention, amplification of an idea must come with an explicit attribution to the person who first introduced the idea. Amplification leads to positive outcomes such as ideas being rated higher, and both the original voicer and amplifier being seen as having higher status^40^. On the other hand, idea stealing leads to negative outcomes for the idea stealer such that those who are perceived to be stealing an idea are judged to have worse character than those who steal money, and individuals are often less willing to offer them co-worker support^9^. Considering the consequences of both idea-stealing and amplifying behaviours, it is apparent that in group settings it is important to explicitly give credit to the person whose idea is restated.

Future work on this phenomenon would benefit from using samples that are more diverse in terms of age, education, work experience, and industry, including people who are non-WEIRD^40^, in order to investigate cultural, societal, and individual differences in how attentive individuals are to idea stealing. In doing so, research could compare the rate of social inattentional blindness across different demographic groups and social environments. For example, one limitation of this paper is that the majority of the sample was Asian but none of the team members were Asian, while analyses showed that there were no differences between noticing across ethnicity (see supplementary materials), future research examining the intersection between participants ethnicity and team member ethnicity would be valuable. Further, it would be valuable to examine how the race and gender of the idea stealer or original idea sharer affect social inattentional blindness.

This paper’s contributions should be viewed in light of its strengths and limitations. The use of VR in this paper is a methodological strength, as it enabled a tightly controlled and extremely realistic test of the research question. This goes beyond past work on inattentional blindness that has primarily used video-based materials which unnaturally restricts the field of vision for the participants^1^. Further, this paper also goes beyond past work using the “who said what” paradigm which has primarily used abstract stimuli (i.e., pictures of people with quotations attached to them, or voice recordings^15^). By immersing individuals in the environment, this study benefited from the social presence created in VR^37^, which has been shown to be critical in generating realistic estimates of how individuals pay attention to social stimuli^16^. Nevertheless, there are still limits to using video-based VR as a research tool as it does not allow individuals to interact with their environment, other than having agency over where participants choose to fixate their attention.

Another limitation of this paper is the lack of a direct effect of diversity on noticing. While there is a numerical difference of 6% more participants noticing in the diverse group, the only statistically significant difference was indirectly through serial mediation (see Supplementary Materials). In interpreting this null effect, it is important to note that although the perceived diversity of the “diverse” experimental condition team was higher than that of the “homogeneous” experimental condition team, both were rated as below the scale midpoint (see Table 1). Further, although the diverse team objectively was diverse in terms of contexts with minimal participation of people who are not White men (e.g., boards and top business executives in the Americas and Europe), the diverse group was still not as diverse as the participant sample. Finally, this paper also manipulated racial *and* gender diversity simultaneously, instead of manipulating gender *or* racial diversity in separate conditions. Therefore, we cannot rule out whether the effects of gender and racial diversity are different, which would be an interesting domain for future research to consider. We encourage future research examining the effects of diversity on noticing idea stealing in more diverse groups (i.e., those that better represent participant and population samples).

## Method

In this study, we developed a VR-based social experiment in which participants were immersed in a two-minute idea-generation meeting and tasked with identifying the best solution, which was embedded within a larger 20-min decision-making meeting. Ethical approval for this study was obtained from Rice University’s Institutional Review Board (IRB) and the experiment was performed in accordance with relevant guidelines and regulations. All participants were over 18 and provided informed consent before taking part in the experiment and were free to leave the study at any point.

During the meeting, one meeting participant repeated the idea of another participant, presenting it as his own. Participants were randomly assigned to one of two experimental conditions, one in which all four of the group members were White men (homogeneous), and another in which the group members were two White men, one Black man, and one White woman (diverse). The primary outcome of interest was whether participants correctly identified the first contributor of the idea—the person whose idea was stolen.

### Participants

A total of 154 participants were recruited from a major university in the United States. The average age of participants was 20.36 years old (SD = 1.90) and 56.76% self-identified as women. The sample was majority Asian (42.57%), White (27.03%), and Hispanic (22.30%), with fewer participants identifying as Black (6.49%) or Other (1.95%). Most participants (89.19%) had experience working in a team in which they were asked to make meaningful decisions (similar to the VR scenario). Further, 85.71% had over 3 months of work experience and 40.14% had over 3 years of work experience.

### Procedure

**Figure 3:**
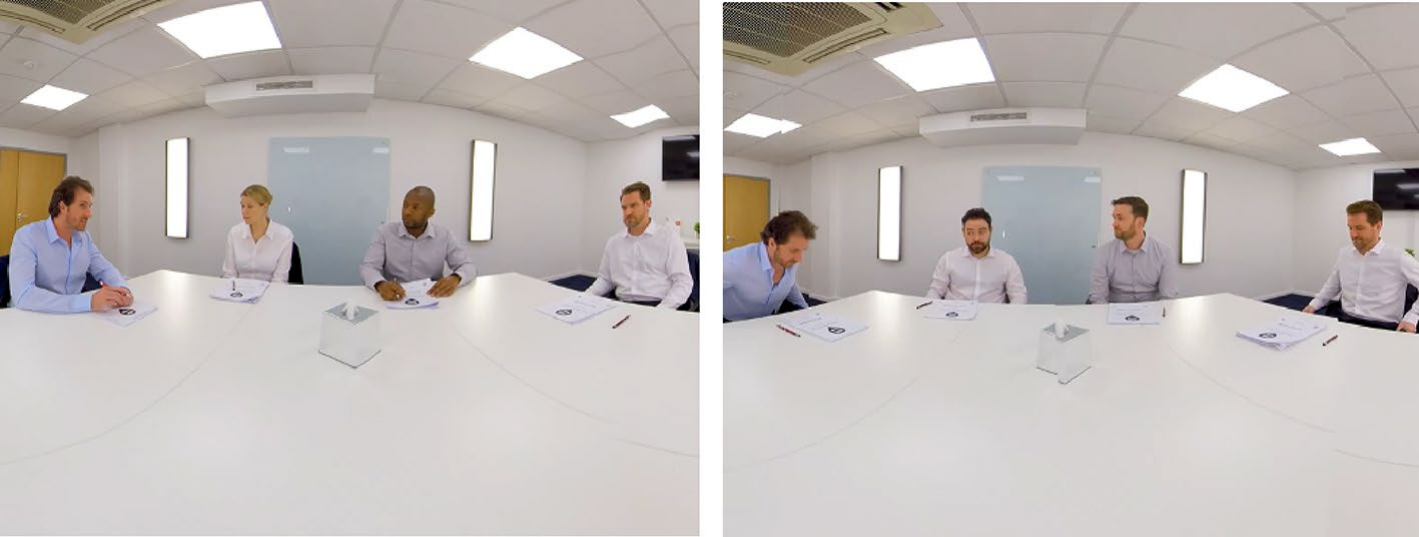
Two-dimensional representation of the VR environment. The image on the right is the homogenous group, the image on the left is the diverse group. Note, within VR, participants field of view is 110°, allowing them to see both central team members when looking forward, and only the person on the right when looking right and only the person on the left when looking left. The person on the far right was the idea sharer, the person on the far left was the idea stealer. Further, the individuals in these images were paid actors, who provided consent for their images to be used in this VR social experiment.

This study used a between-subjects design, randomly assigning individuals to one of two experimental conditions. In the homogenous condition, participants were seated in a room of four decision makers who were all White men, and in the diversity condition, participants were seated in a room of four decision makers of which two were White men, one was a Black man, and one was a White woman (see Fig. 3).

Participants viewed a 20-min VR vignette of a decision-making scenario called the Market Consulting Group (MCG). The VR vignette is a social decision-making scenario in which a team makes decisions about a business venture. This vignette is based on a widely used negotiation case (Towers Market^27^) in which shop vendors (e.g., grocery, florist, liquor store) make a series of decisions about a collective (physical) marketplace they will be joining, called Towers Market. In this version of the case, the decision makers are not the vendors, but a group seeking to make an optimal decision given each of the vendors’ concerns. For the script segment including the idea stealing scene, see Appendix 1.

The VR-scenario had a short introduction and conclusion on either side of four separate decision-making scenes (2–5 min each). All four decision-making scenes required participants to listen to the information shared to make a decision. The final decision-making scene contained the case of idea stealing. Below we focus on the final scene in detail, which was used to test this study’s hypotheses.

In the scene in which the idea stealing occurred, participants decided what location the market would be built in. The options generated by the group were the riverside, industrial site, or city center. In the scene, all three ideas are shared. First, the team member on the far right shares the idea of building it by the riverside, this idea is then not discussed further. Next, two of the team members in the center share the idea to build the market at the industrial site and city center, but these ideas are dismissed by the rest of the team after discussion. Finally, the person on the far left shares the riverside idea *again* (i.e., steals it from the person on the far right). The team then all endorses this idea. See Appendix 1 for the script. After the scene is complete, participants choose which location the market should be built at (riverside, industrial site, or city center; note 99.33% choose the riverside), then participants are asked who shared each idea first.

During the scenes, participants also responded to thought probes to indicate whether they were paying attention or mind-wandering. These probes appeared every 22 to 28 seconds. Participants saw the question, “Which of the following two options best describes where your attention was focused immediately before this question appeared?”. The two response options they had were (a) On-Task (listening to team members, watching team members’ reactions) or (b) Off-Task (mind-wandering, distracted, mind was blank). After the participant answered the question, the video would restart. For the location decision, there were a total of five probes.

After completing the VR scenario, participants answered a series of questions. This included perceptions of the team, psychometric measures of attention, and demographics.

### Vignette development

The script for this scenario was created by the first two authors and another academic (see acknowledgments), these three researchers formed the paradigm development team in collaboration with a VR team, and a professional script writer. Business school professors who study group decision making and boardroom meetings provided feedback on the script, which was adjusted accordingly.

Actors were recruited through a professional recruiting agency and the paradigm development team put significant effort into identifying actors that were similar aside from demographic differences where noted. All actors were recruited from the same age range, all spoke using a neutral American accent, and all wore business attire (See Fig. 1).

All actors attended 3 rehearsal days in which they worked with a director. The directorial guidance was written by the first author and had actors focus on delivering information clearly and concisely and toning down the character’s personality. Particular attention was given to actors playing the same role to match the prosody of their voice, the personality of the character, and body language. The vignette was then filmed over three days using a professional filming crew provided by the VR Team.

Two identical vignettes were recorded, the first using 4 white men as the actors (homogenous team), the second using 2 white men, 1 black man, and 1 white woman (diverse team). We chose to have one group that was 4 white men because we understand that, in the US, this is the prototypical view of what a non-diverse, homogenous, work team would look like. This is based on figures indicating that white men are more likely to hold senior positions in an organization^41^, and documented biases favoring white men in organizational settings^42,43^. For the diverse group, we wanted to manipulate both gender and racial diversity (relative to the four white men group) but also keep the gender and race of the “original idea sharer” and the “idea stealer” the same. Therefore, we opted for the other two members of the team to be a white woman and a black man.

### Measures

#### Location decision

Participants decided which location would be best for Towers Market by selecting one of the “Riverside”, “City Center”, or “Industrial Site”. All participants except 1 (99.33%) chose the riverside as the team verbally agreed this was the best choice in the meeting.

#### Noticing idea stealing

Participants were asked to indicate “Who said the location idea first?” and choose between each of the team members (pictures of each team member arranged in the order they sat around the table were included to facilitate identifying the team members). This variable was operationalized as correctly selecting the original idea generator as the person who shared the location idea first being coded as “1” and all other responses being coded as “0”. Thus, the reverse of noticing idea stealing was our measure of inattentional blindness.

#### Idea credit

Participants reported “Who deserves credit for the riverside idea?”, choosing between each of the team members (again, pictures were included).

#### Perceived team diversity

Participants responded to a one-item question, indicating whether they agreed with the statement that the team was “diverse” (1 “Strongly Disagree” to 7 “Strongly Agree”).

#### General attentiveness

A 4-item scale measuring attentional engagement was used as a measure of general attentiveness^28^. The original form scale was adapted to be a state measure, and the focus of the measure was shifted from “work” to the “interview”. Sample items include, “I spent a lot of time paying attention to the interview,” and “I concentrated a lot on the interview”. Items were rated on a Likert scale (1 “Strongly Disagree” to 7 “Strongly Agree”): α = 0.94.

#### General inattentiveness

A 5-item scale measuring off-task thought measured general (in)attentiveness^29^. The scale has been adapted to be a state measure, and the focus of the measure has been shifted from the “task” to the “interview”. Sample items include, “I took ‘mental breaks’ during the interview” and “I daydreamed while listening to the interview”. Items were rated on a Likert scale (1 “Strongly Disagree” to 7 “Strongly Agree”): α = 0.74.

#### Perceived status

Perceived status was measured using a three-item scale^30^. The scale was adapted to the team level and included the items, “This team has a good reputation among those they work with.”, “Others look up to this team because they are good at their job.” and “Others seek this team's opinion because they respect them.” Items were rated on a Likert scale (1 “Strongly Disagree” to 7 “Strongly Agree”): α = 0.85.

### Supplementary Information

Supplementary Information 1.

Supplementary Information 2.

## Data Availability

All data generated or analysed during this study are included in this published article are available at https://osf.io/gbwsy/.
