# Supplementary material for: Social inattentional blindness to idea stealing in meetings
Source: Sci Rep. 2024 Apr 5;14:8060. doi: 10.1038/s41598-024-56905-6 (PMC10997580; doi:10.1038/s41598-024-56905-6)
Supplement: Supplementary file 1 — Supplementary Information 1. [file 41598_2024_56905_MOESM1_ESM.docx]

**Supplementary Materials for:**

**Social Inattentional Blindness to Idea Stealing in Meetings**

Theodore C. Masters-Waage^*1,2^, Zoe Kinias^3^, Jazmin Argueta-Rivera^4^, Dillon Stewart^4^, Rachel Ivany^4^, Eden King^4^, Mikki Hebl^4^

^1^INSEAD, ^2^University of Houston, ^3^Western University, ^4^Rice University

*Corresponding Author: Theodore C. Masters-Waage ([tmasters@central.uh.edu](mailto:tmasters@central.uh.edu))

**Details:**

This supplement includes additional analyses of the data not reported in the main manuscript along with further explanation of the scenes included in the VR experience (see Masters-Waage (2022) - doctoral dissertation - for more details)

- Additional Analyses (p2-p3)
- Figure S1 (p4)
- Table S1 (p5)
- Additional Decision Scenes (p6-p7)

**Additional Analyses**

To supplement the analyses in the main manuscript additional analysis were conducted to examine exploratory hypotheses. Specifically, two analyses were conducted, the first focusing on the effects of participant race and gender on noticing and the second on the indirect effect of the diversity condition on noticing via perceived team status.

**Effect of Participant Gender and Race on Noticing**

To examine possible relationships between participant demographics and noticing we ran a series of exploratory correlations (see Table S1). Noticing was coded as “1” for participants who noticed the idea stealing and “0” if not. Gender was coded as “1” for women and “0” for men; note, only one participant responded “prefer not to say” for gender and was not included in the correlations. For the coding of race/ethnicity, we generated a series of dummy variables. First, given that the majority of the sample was Asian, we created a dummy variable in which Asian participants were coded as “1” and all other participants as “0”. Second, we created a dummy variable indicating if participants' ethnicity/race was represented in the team (“1” = White or Black) or if it was not (“0” = Asian, Hispanic, Native Hawaiian, or Pacific Islander). Third, given that the majority of team members in the VR simulation were White, we created a dummy variable indicating if participants were White “1” or not “0”. All correlation coefficients were non-significant indicating that demographics did not play a major role in determining noticing (see Table S1).

**Indirect Effect of Diversity Condition on Noticing via Serial Mediation**

Given there was an effect of status but no direct effect of diversity, on an exploratory basis, we examined whether there was an indirect effect of diversity on noticing idea stealing via perceived (team) status. We conducted a serial mediation model (see Figure S1) to examine whether team diversity increased noticing through a sequential process of increasing perceived diversity, which in turn increased perceived team status, which in turn increased general attentiveness, and ultimately increased noticing. Results found no direct effect of team diversity on noticing (*b =* .87, *SE =* .50*,* p > .05), nor was there a significant total effect (*b =* -.47, *SE =* .39*,* p > .05]). However, we found evidence for a three-stage serial mediation model with experimentally manipulated team diversity affecting noticing via increased perceived diversity, perceived status, and general attentiveness (*b* = .06, *SE* = .05, p < .05). In sum, this provides evidence for an indirect effect of team diversity on reducing social inattentional blindness through a serial mediation process.

**Figure S1.** Theoretical Model for serial mediation.


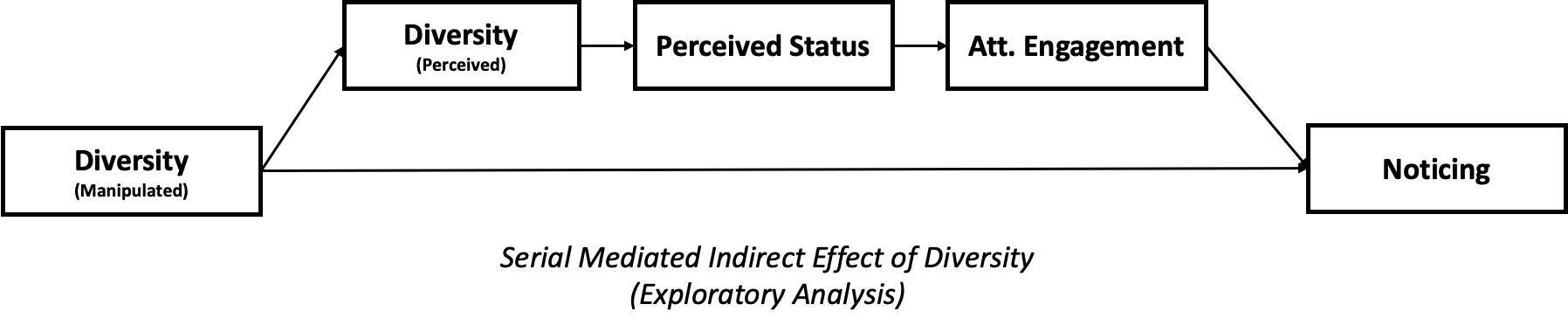


**Table S1.** Binary correlations between noticing and demographic variables.

|  | 1 | 2 | 3 | 4 |
| --- | --- | --- | --- | --- |
| 1. Noticing | - |  |  |  |
| 2. Gender | .07 | - |  |  |
| 3. Asian | -.03 | .08 | - |  |
| 4. Represented in Team | -.08 | .01 | -.57^***^ | - |
| 5. White | -.10 | -.04 | -.52^***^ | .91^***^ |

**Notes.** Variable coding is as described: Noticing coded as 1 “Participants correctly noticed the original idea sharer” or 0 “they did not”, Asian coded as “1” and all other participants as “0”, Represented in Team in the team coded as “1” (White or Black) or if they were not “0” (Asian, Hispanic, Native Hawaiian, or Pacific Islander), and White coded as “1” if the participants was white or “0” if not.

^***^p<.001**,** ^**^p<.01**,** ^*^p<.05**,** ^+^p<.10

**Additional Decision Scenarios**

Along with the location decision reported in the main manuscript, there are also three other decisions that were included in the VR experience. This data was not analyzed but are described in the dissertation by Masters-Waage and repeated below for convenience^1^.

**Balanced Decisions (Advertising).**

In the balanced decision, there were an equal number of social decision cues in favor of the two-response option (advertise: collectively vs. individually). Therefore, both responses are viewed as equivalent. In this decision, one focal actor shares all the social decision cues for one response option (Focal Actor 1: advertise collectively) and the other focal actor shared all the social decision cues for the other optimal decision (Focal Actor 2 (gender-manipulated actor): advertise individually). For example, if the decision is between apples and oranges, one focal actor would share all cues in favor of apples and the other all cues in favor of oranges. The balanced decision has 6 cues. See the image below for a pictorial representation using “New York” and “Los Angeles” as example decision outcomes:


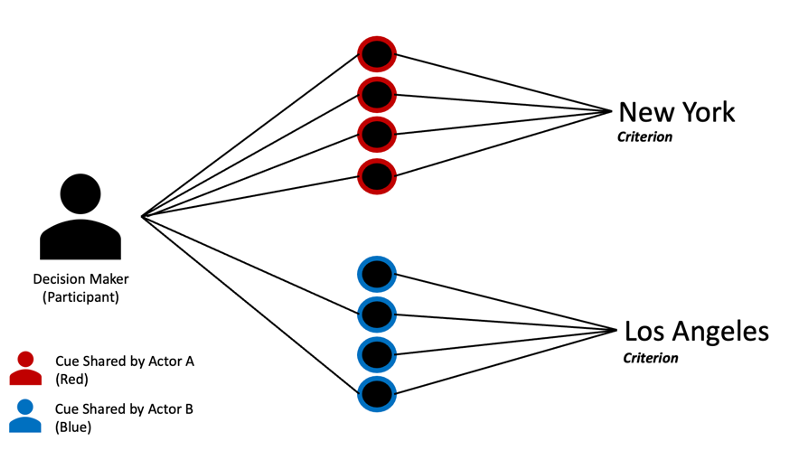


**Unbalanced Decision (Clerks).**

In the unbalanced decision, there was an optimal outcome. This is because there were 4 cues in favor of “hiring clerks individually” and 3 cues in favor of “hiring clerks collectively”. Therefore, if we assume that the cues are weight equally and individuals use a tallying heuristic^2^ then participants who hear all the information (or at least listen in a non-biased fashion) would be more likely to choose the prior option. See the image below for a pictorial representation using “New York” and “Los Angeles” as example decision outcomes:


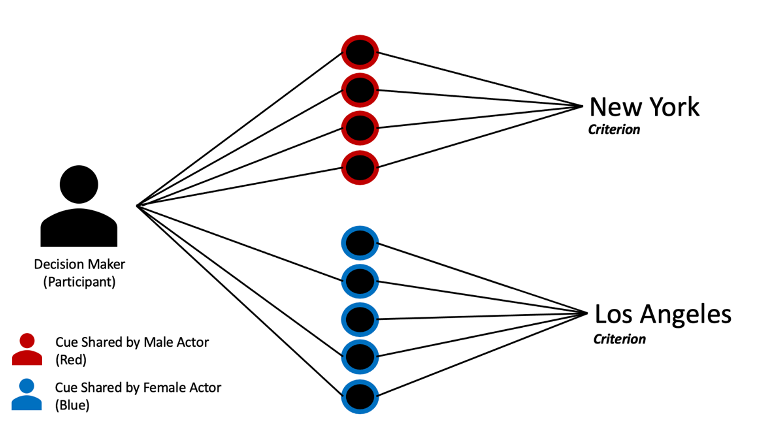


**Hidden Profile Decision (Maintenance).**

In the hidden profile decision, there is an optimal choice. This is because whilst there are 4 cues in favor of doing maintenance collectively and only 2 cues for doing it individually, one of the cues for doing maintenance individually is a critical cue. This critical cue is that “one vendor will drop out if the maintenance is done collectively”, this cue is critical because the participants have been informed that if any vendor drops out then the whole market will fail. All the optimal choice cues are shared by one focal actor (including the critical cue) and all the suboptimal choice cues are shared by the other focal actor. See the image below for a pictorial representation using “New York” and “Los Angeles” as example decision outcomes:


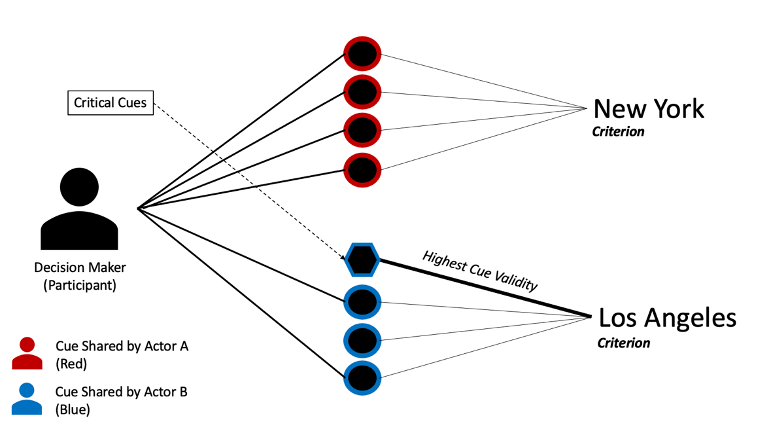


**References**

1. Masters-Waage, T. C. Social attention in realistic work environments. (Singapore Management University, 2022).

2. Gigerenzer, G., Reb, J. & Luan, S. Smart heuristics for individuals, teams, and organizations. *Annu. Rev. Organ. Psychol. Organ. Behav.* **9**, 171–198 (2022).
