# Supplementary material for: Social inattentional blindness to idea stealing in meetings
Source: Sci Rep. 2024 Apr 5;14:8060. doi: 10.1038/s41598-024-56905-6 (PMC10997580; doi:10.1038/s41598-024-56905-6)
Supplement: Supplementary file 2 — Supplementary Information 2. [file 41598_2024_56905_MOESM2_ESM.docx]

# **Appendix 1**

# LOCATION DECSION SCRIPT

LOUIS

Shall we take a look at locations?

# JORDAN

(going through papers) Let's a have a look.

# LOUIS

What are the key indicators here? Cost, parking and location?

# KEN

Yeah.

# JORDAN

I say we propose options and then take them apart a little.

# KEN

Nice. I mean there are so many here it would take us ages to go through each of them.

# LOUIS

Have you been through them all?

Ken looks guilty.

# JORDAN

I mean I'm surprised they are leaving this so late.

# KEN

It's all about flexibility.

# LOUIS

And the same design will work in all these locations.

# KEN

(joking)

All thousand of them. (beat)

The first option I can think of is the City Centre site.

# LOUIS

That's got superb access, so we would get a lot of footfall.

# SAM

It's has high rent, though.

# JORDAN

And we have to consider that people won't be able to carry their stuff home easily. They'll be restricted in their purchases by what they can take on public transport.

**Original Idea Sharing**

# SAM

Another option is the riverside. It offers low rent and has parking options.

# LOUIS

Isn't there another market opening in the City Centre?

# KEN

Not that I know of.

# JORDAN

There used to be one, I know that. It closed down.

# LOUIS

I thought I saw developments. That said the city always has development.

# SAM

Its starting to feel like the only permanent structures are the cranes.

# LOUIS

Alright, what about the industrial site?

# JORDAN

The businesses around there are very different, I feel.

# KEN

I see, it's maybe not the right vibe.

# SAM

But the rent is cheap.

# LOUIS

It is a lot cheaper. Shame it's so far from the city centre.

# KEN

A lot of these locations aren't viable.

Pause.

# LOUIS

Two are in residential areas.

# JORDAN

Getting planning permission there would be a nightmare.

# LOUIS

Especially as they would want to be building morning and night.

# SAM

Wouldn't want that happening near my home.

**Idea Stealing**

# JORDAN

(Has an idea)

Hey, I don’t think we’ve discussed the Riverside. You might have missed it, it was deep in the pile. It might be what we’re looking for.

# LOUIS

The Riverside... oh, yeah.

# JORDAN

It has space for parking.

# KEN

Also, there is a residential space being built nearby.

# LOUIS

And they are offering low rent because the development is new. I'm sure we'd get a good deal.

# JORDAN

Great. Sounds like the Riverside to me.

# SAM

I think it's an obvious choice.

# LOUIS

Nice work everyone. Choice made.

# KEN

(to Participant)

Hey, do you mind if I ask you a question.
